# Wave-current and wind climate in the remote foreshore of a non-tidal sea in view of field investigations

**DOI:** 10.1038/s41598-025-12145-w

**Published:** 2025-07-21

**Authors:** Magdalena Stella-Bogusz, Rafał Ostrowski

**Affiliations:** Institute of Hydro-Engineering, Polish Academy of Sciences, ul. Kościerska 7, 80-328 Gdańsk, Poland

**Keywords:** South Baltic Sea, Coastal zone, Field measurements, Waves, Wind-driven current, Physical oceanography, Fluid dynamics, Physical oceanography

## Abstract

The paper presents results of around 14-month long field survey monitoring wave-current and wind parameters at a remote foreshore location representative of the south Baltic Sea. Water surface elevations and superficial flow velocities were measured using a wave-current buoy moored at a depth of 18 m, about 2.8 km from the shoreline, while wind data were collected with an anemometer mounted on a nearby land mast and then recalculated to obtain the wind velocity over the sea. The analysis shows that the predominating direction of wave propagation is from W to E while the wind-driven currents are mostly directed from WSW to ENE. The extreme wave heights amounted to *H*_*s*_ = 4.36 m and *H*_*max*_ = 7.33 m while the maximum measured superficial flow velocity amounted to *u*_*meas*_ = 1.09 m/s. Such strong superficial currents had never been recorded in the region. The extreme wind speed recorded in the survey period was equal to *W*_*sea*_ = 19.93 m/s. The wind-driven superficial flow velocity was successfully modelled by a simple approach yielding a satisfactory agreement with the measured quantities. The presented novel findings have been achieved using a multi-method approach, comprising viewing of the time series, rose plots and Dot Products.

## Introduction

While the nearshore surf zone of a non-tidal sea is dominated by phenomena related to wave energy dissipation, such as wave breaking and wave-driven currents^1^, wave propagation in regions located further from the shoreline is accompanied mainly by currents typically observed in deeper sea, e.g. by wind-induced flows^2,3^.

In the south Baltic coastal regions, the remote foreshore zone stretches roughly to the depth of 20 m^4^. The shoreward boundary of this zone coincides with a so-called depth of closure, i.e. the depth beyond which no significant wave-induced sea bed changes take place^5^. According to the investigations by Ostrowski and Stella^6^, motion of sandy bottom sediments is still possible at depths greater than the depth of closure because of the synergic impact of storm waves and strong wind-driven currents. The sediment transport rate depends on the bed shear stresses imposed on the sea bottom by water motion taking place within complicated nonlinear wave-current interactions representing the influence of the wave bed boundary layer on the steady flow^7^. Investigations concerning features of waves and currents in the remote foreshore, leading to a better understanding and a more precise description of the wave-current climate in this region, are therefore crucial in a reliable predictive modelling of lithodynamic and morphodynamic processes occurring in a close vicinity of the surf zone.

In the early 1990s, long-term wave measurements were initiated in the region under consideration. The surveys were carried out using wave buoys moored usually at water depths of 15–20 m. Since 2012, the buoys have been deployed at a depth of 18 m, corresponding to an offshore distance of about 2.8 km^8,9^.

Recently, joint measurements of free surface elevations and superficial flow velocities have become possible due to the application of a new device. A simultaneous monitoring of waves and currents at the same location can shed more light on the characteristics of hydrodynamic phenomena in the remote foreshore. In particular, the knowledge of wave-current parameters and wind conditions can be used in identification of swell and wind waves, wind-driven currents and other types of currents occurring beyond the surf zone of a non-tidal sea.

The paper deals with the abovementioned hydro-meteorological processes, investigated within a long-term field survey, presenting features of the wave-current climate of the south Baltic coastal zone. The significance of this research is augmented by the region’s strategic role in Poland’s energy transition. Just a few kilometers northeast, the Baltic Power—Poland’s first offshore wind farm has entered construction, aiming to generate around 1.14 GW by 2026 and supply power to approximately 1.5 million households^10^. Simultaneously, a few kilometers west, preparatory work is on track for Poland’s first coastal nuclear power plant at Lubiatowo–Kopalino in Pomerania^11^. Thus, understanding wave, current, and wind dynamics in this region is crucial—not just scientifically, but for the design and safe operation of these major low-emission energy facilities.

## Study site and field measurements

**Fig. 1:**
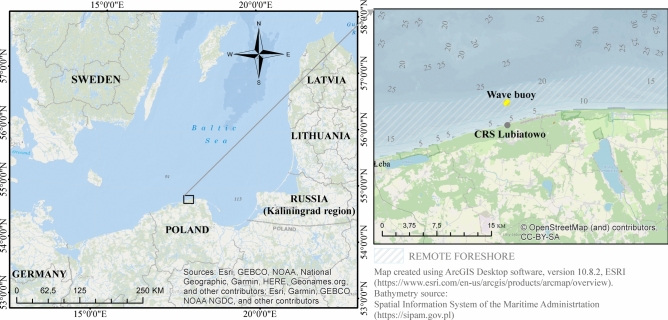
Location of CRS Lubiatowo on the south Baltic coast.

The Coastal Research Station (CRS) in Lubiatowo, Poland, is a field facility operated by the Institute of Hydro-Engineering of the Polish Academy of Sciences (IBW PAN). Located on the south Baltic coast (Fig. 1), it is the only one of this kind in Poland and one of few worldwide, carrying out coastal research in undisturbed natural conditions. Established in 1968, CRS Lubiatowo has provided a rich database and findings related to hydrodynamic, lithodynamic and morphodynamic processes observed in both the nearshore zone and more remote coastal regions [see e.g. Refs.^3,8,12–15^].

Since the 1990s, water surface elevations in the remote foreshore have been measured using mostly Directional Waverider buoys Mk. II and Mk. III (produced by Datawell B.V., the Netherlands). Analyses of archival data show that the mean wave period *T*_*mean*_ amounts to 3–7.5 s, reaching 9 s at maximum. The most frequent waves, occurring for 47% of time, have a mean height *H*_*mean*_ of 0.5–1.5 m. Extreme wave conditions were recorded at a water depth of 18 m during the hurricane Xavier on Dec. 6, 2013, with the maximum wave height *H*_*max*_ = 7.80 m, the maximum significant wave height *H*_*s*_ = 4.42 m and the corresponding significant wave period *T*_*s*_ = 9.3 s.

Flow velocities in the remote foreshore of this region were investigated in situ once only, during a short field campaign from Apr. 26 to Jun. 30, 2014. The measuring device, namely an acoustic current meter, was installed 2.7 km offshore, at the position 54° 50.48′ N, 17° 53.09′ E, where the water depth amounted to *h* = 17 m. Results of those measurements^2^ revealed a superficial current with a maximum velocity of 0.58 m/s. This extreme value was recorded during a relatively heavy storm with the significant wave height *H*_*s*_ = 2.75 m.

The measurements at CRS Lubiatowo described in the present paper were carried out using a Directional Waverider buoy with an Acoustic Current Meter option (DWR4/ACM for short). Its features are as follows: diameter of 0.7 m, cunifer hull with an anti-spin triangle and radar reflector; sea surface temperature meter, GPS device, 1 Gb datalogger, led flashlight and a battery life of 16 months; data transmission and tracking via GSM Internet (dial-in or GPRS). The buoy was moored at a depth of 18 m, about 2.8 km from the shoreline, at the position 54° 50.34′ N, 17° 50.33′ E. The measurements covered the time period from Nov. 26, 2022 to Jan. 30, 2024.

Parameters of water surface elevation (heave and direction) were obtained by DWR4/ACM from a multidirectional acceleration sensor while parameters of the current (speed and direction) were measured by three acoustic-Doppler transducers within a cell size of 0.4 m to 1.1 m from the water surface. The accuracy with respect to water surface elevation amounted to 1% of the measured value. The current meter accuracy was equal to 1% of the measured value ± 2 cm for velocity and 0.4° to 2° (depending on latitude), typically 0.5°, for direction. The output data rate for water surface elevations was 2.56 Hz, while the current meter data were updated every 10 min.

Simultaneously, wind velocities and directions were measured at CRS Lubiatowo with an anemometer mounted on a land mast, about 150 m from the shoreline, at the position 54° 48.70′ N, 17° 50.43′ E. The local wind velocity recorded on land *W*_*land*_ can be recalculated to obtain the wind velocity over the sea according to relationships formulated by Stella^4^:

1 $$W_{sea} = 3.68W_{land}\,\left[ {{\text{m}}/{\text{s}}} \right] \quad {\text{for}}\,W_{land} \le {\text{1 m}}/{\text{s}}$$

2 $$W_{sea} = 1.76W_{land} + 1.92\,\left[ {{\text{m}}/{\text{s}}} \right] \quad {\text{for}}\,W_{land} > {\text{ 1 m}}/{\text{s}}$$

Following Kim et al.^16^, the superficial wind-driven flow velocity *u*_*surface*_, in turn, can be determined from the wind velocity *W*_*sea*_ in the following way^3^:

3 $$u_{surface} = \, 0.0{3}W_{sea} {\text{for}} W_{sea} < {\text{ 8m}}/{\text{s}}$$

4 $$u_{surface} = \, 0.0{35}W_{sea} {\text{for}} W_{sea} \ge {\text{8 m}}/{\text{s}}$$

Results of wave-current and wind measurements provide a new interesting research material on the hydro-meteorological climate of the south Baltic Sea. Interestingly, the survey period included a uniquely extreme storm of January 2024.

## Results

The wave data measured by DWR4/ACM constitute irregular time series of water surface elevation and direction of wave propagation. They were used to determine series of 1-h representative wave parameters, namely the significant wave height *H*_*s*_ (mean of 1/3 of the highest waves in a 1-h wave train), the wave energy peak period *T*_*p*_ (period of a wave having the biggest energy in the wave spectrum), the maximum wave height *H*_*max*_ and the direction of wave propagation *Dir*_*p*_ (corresponding to *T*_*p*_).

According to meteorological standards^17^, instantaneous wind parameters, i.e. speed and direction, are time-averaged over 10 min. Aside from these mean wind parameters, maximum velocities (wind gusts) occurring in 10-min long time periods are stored in the database. As mentioned in the previous section, the flow characteristics (speed and direction) are transmitted by the measuring device with an update rate of 10 min.

**Fig. 2:**
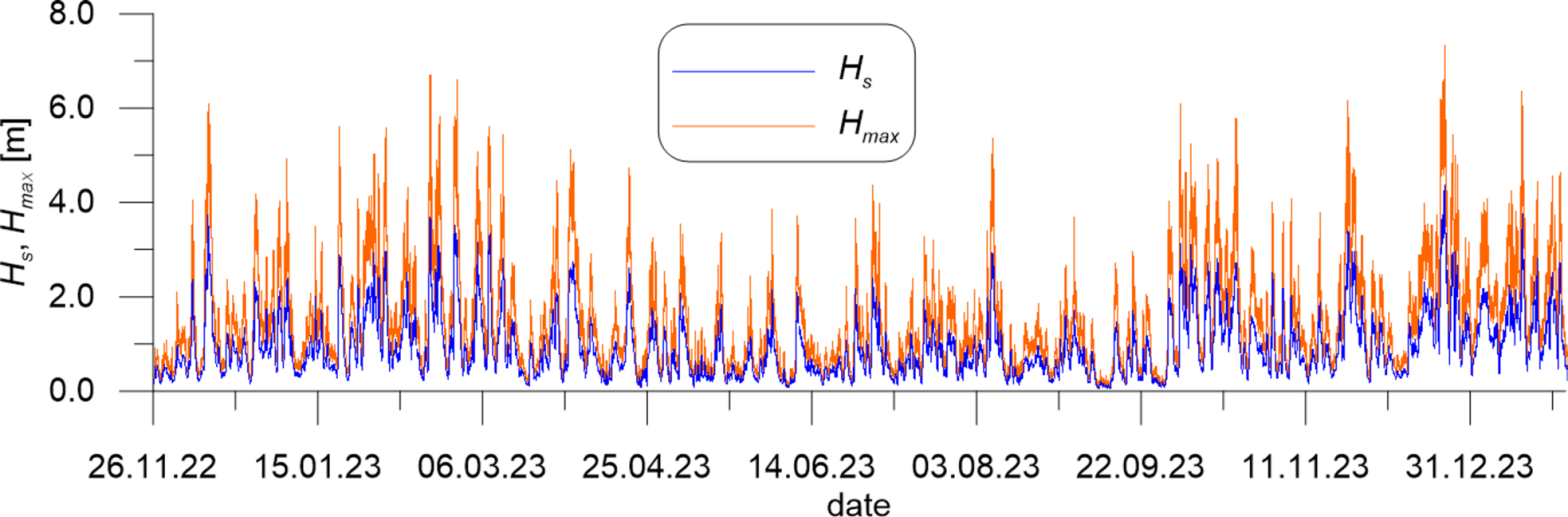
Significant and maximum wave heights *H*_*s*_ and *H*_*max*_ measured at CRS Lubiatowo in the period from Nov. 26, 2022 to Jan. 30, 2024.

**Fig. 3:**
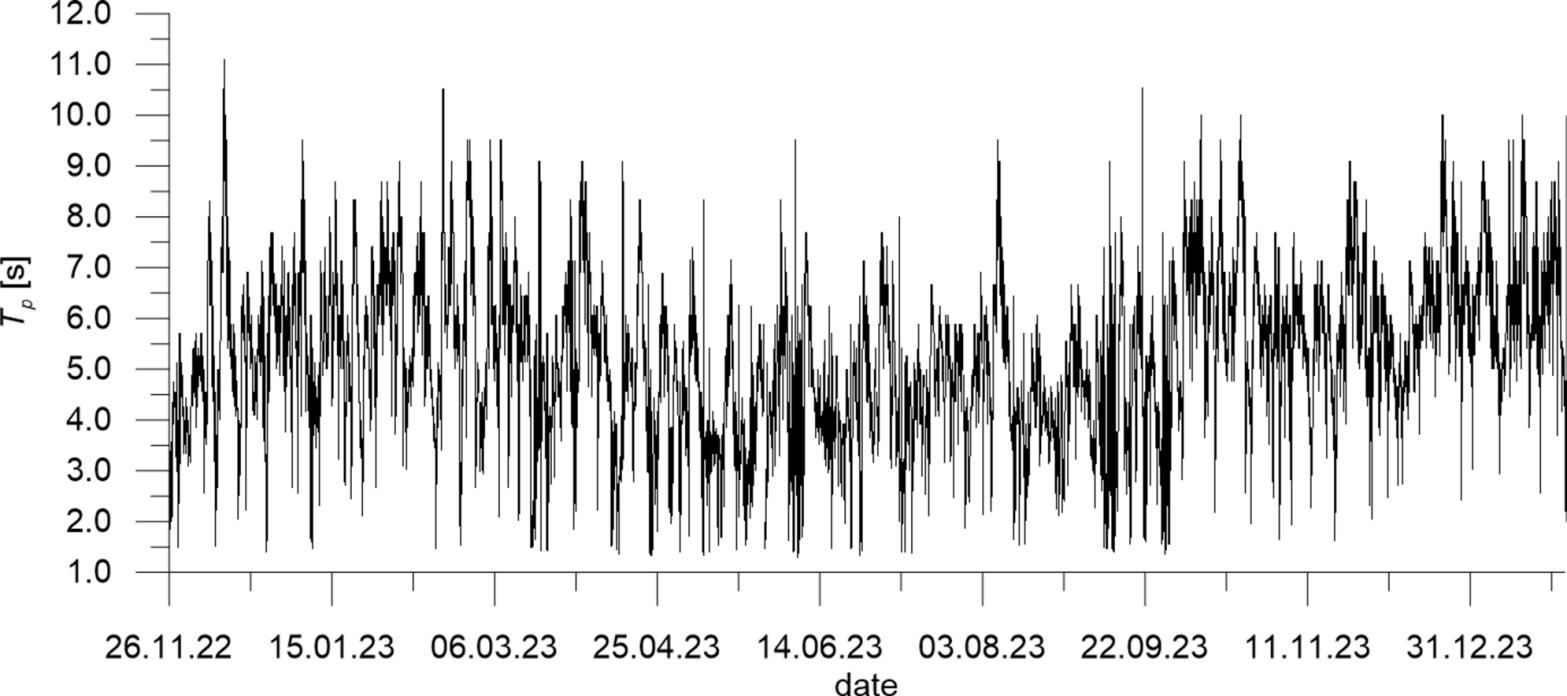
Wave energy peak period *T*_*p*_ measured at CRS Lubiatowo in the period from Nov. 26, 2022 to Jan. 30, 2024.

**Fig. 4:**
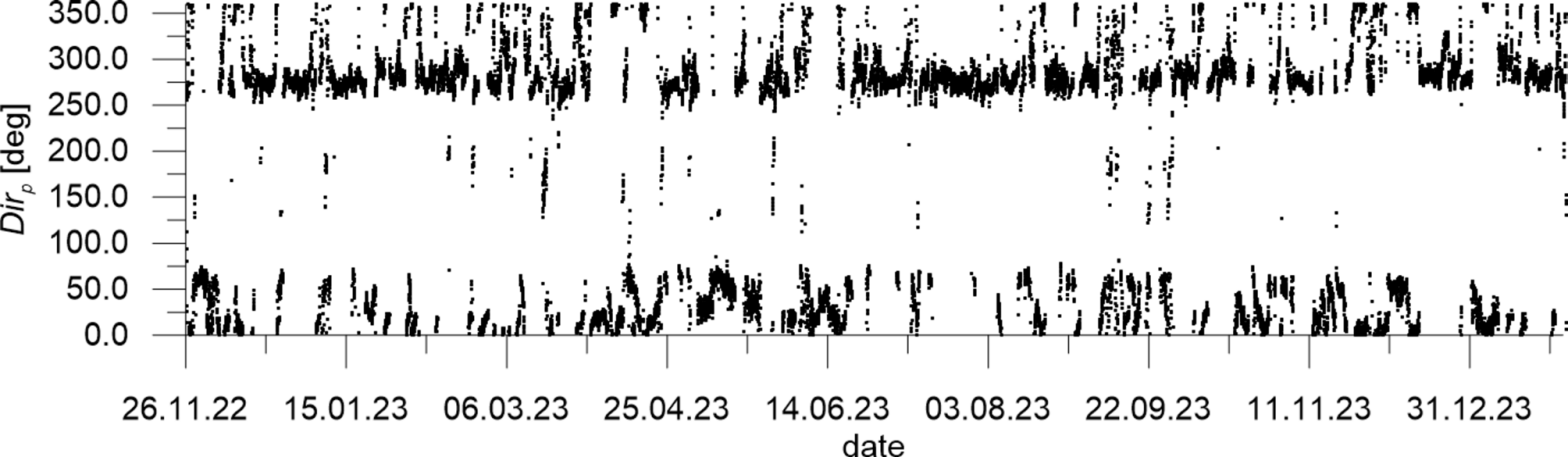
Direction of wave propagation *Dir*_*p*_ (“from”) measured at CRS Lubiatowo in the period from Nov. 26, 2022 to Jan. 30, 2024.

The representative wave parameters *H*_*s*_, *H*_*max*_, *T*_*p*_ and *Dir*_*p*_ measured at CRS Lubiatowo in the period from Nov. 26, 2022 to Jan. 30, 2024 are given in Figs. 2, 3 and 4.

The extreme wave parameters recorded in the survey period were as follows: *H*_*s*_ = 4.36 m, with the corresponding wave period and direction *T*_*p*_ = 9.09 s and *Dir*_*p*_ = 298.55° (on Dec. 23, 2023), *H*_*max*_ = 7.33 m, with the corresponding wave period and direction *T*_*p*_ = 9.52 s and *Dir*_*p*_ = 309.27° (also on Dec. 23, 2023). It can be seen in Fig. 4 that the predominating direction of wave propagation was about 275° (from W to E). This could have been expected in view of earlier investigations on the wave climate in the south Baltic Sea.

**Fig. 5:**
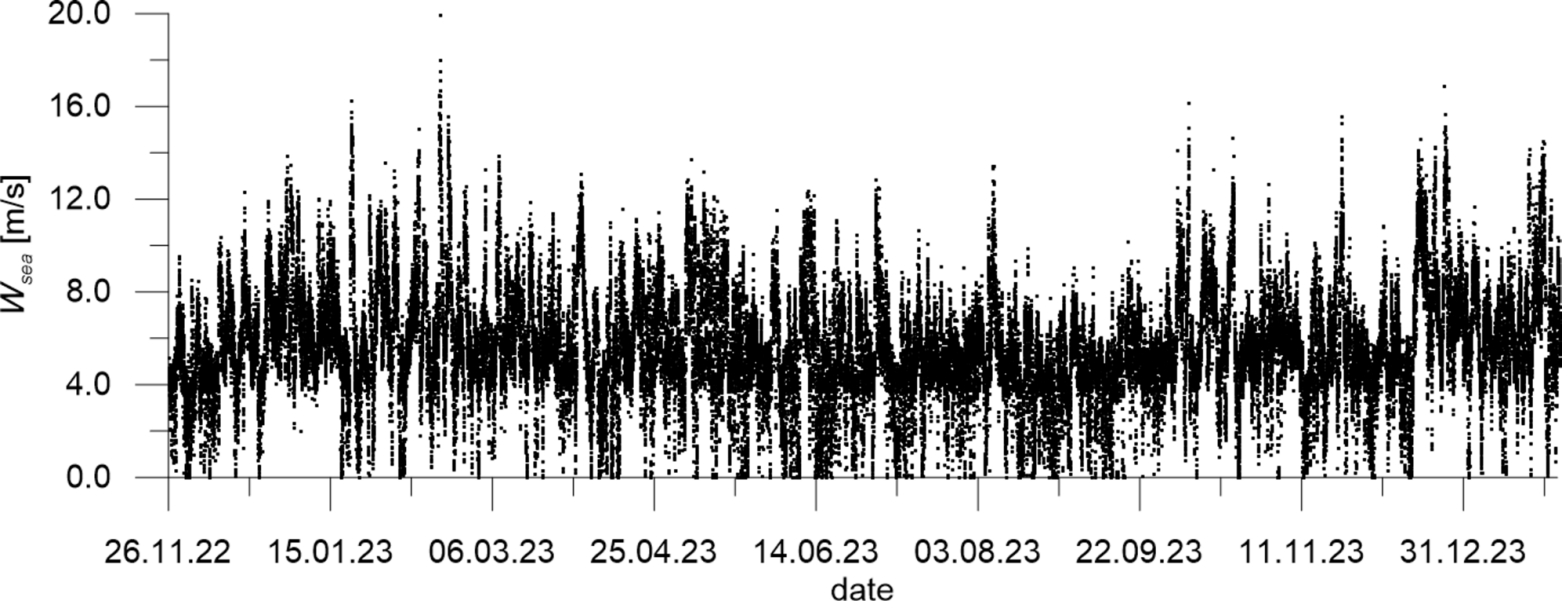
Mean wind velocity over the sea *W*_*sea*_ measured at CRS Lubiatowo in the period from Nov. 26, 2022 to Jan. 30, 2024.

**Fig. 6:**
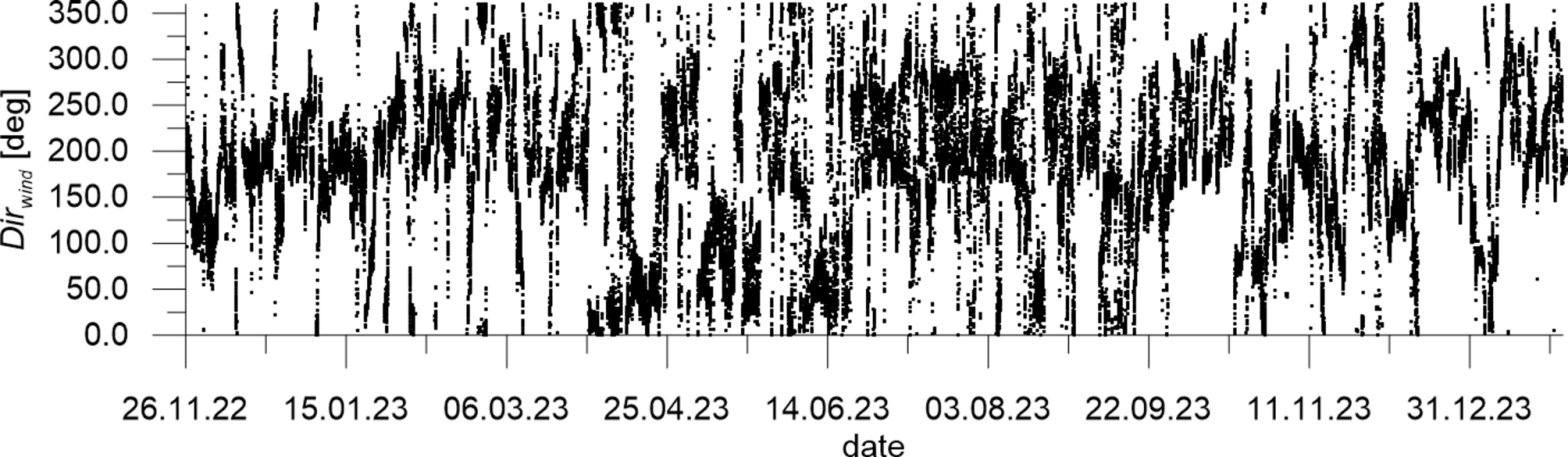
Wind direction *Dir*_*wind*_ (“from”) measured at CRS Lubiatowo in the period from Nov. 26, 2022 to Jan. 30, 2024.

**Fig. 7:**
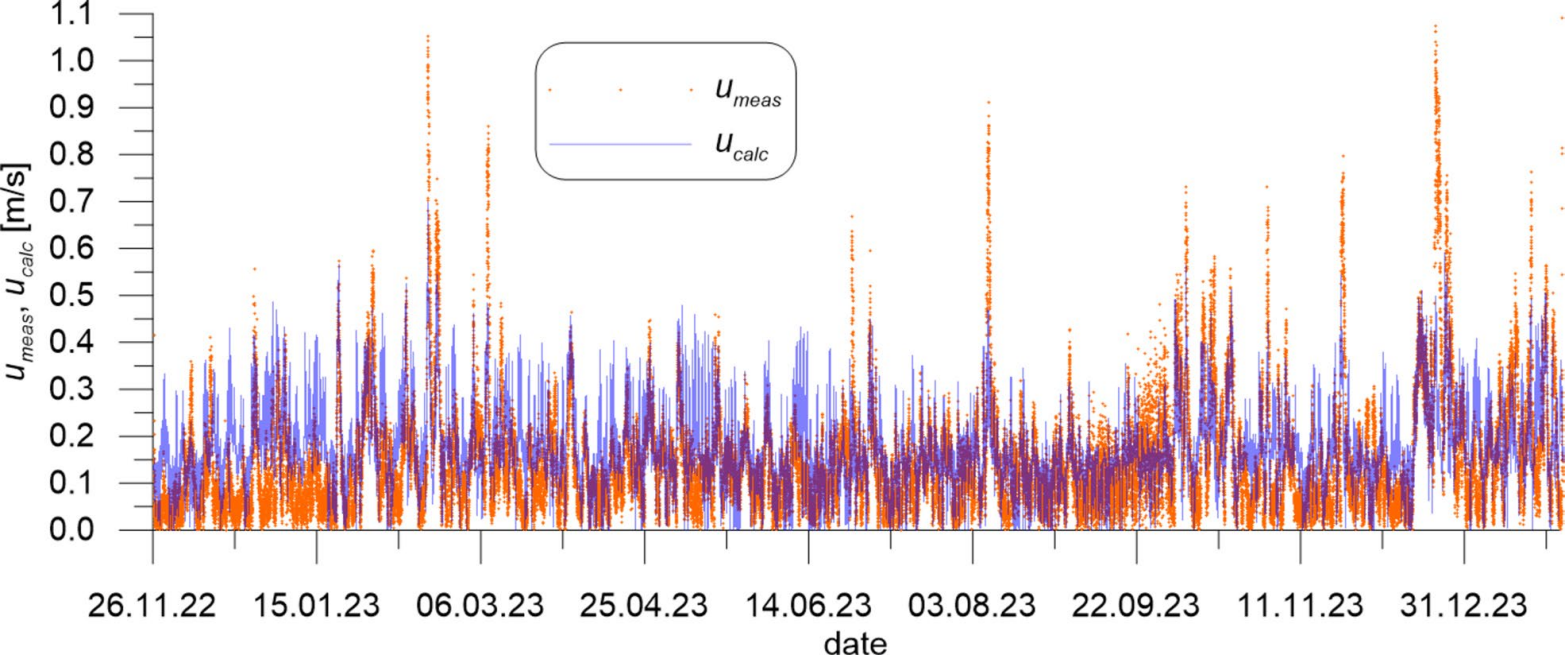
Flow velocity *u*_*meas*_ measured at CRS Lubiatowo in the period from Nov. 26, 2022 to Jan. 30, 2024 and *u*_*calc*_ calculated by Eqs. (3) and (4).

**Fig. 8:**
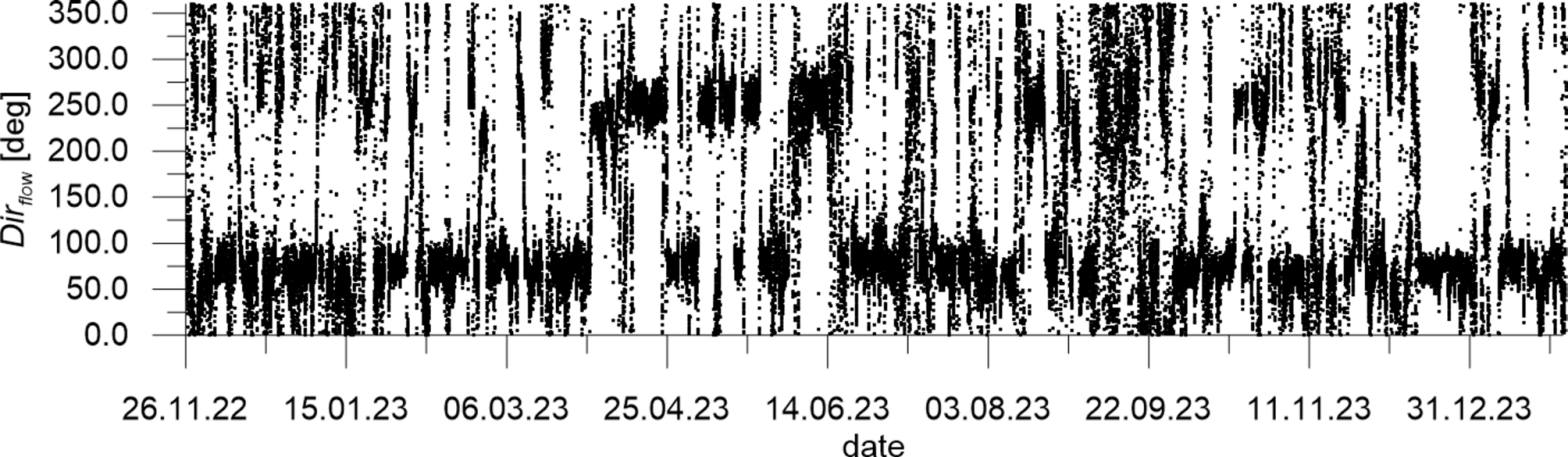
Flow direction *Dir*_*flow*_ (“to”) measured at CRS Lubiatowo in the period from Nov. 26, 2022 to Jan. 30, 2024.

The mean wind velocity over the sea *W*_*sea*_ measured at CRS Lubiatowo in the period from Nov. 26, 2022 to Jan. 30, 2024 and recalculated by formulas (1) and (2), as well as wind direction *Dir*_*wind*_, are given in Figs. 5 and 6, respectively, while the measured flow velocity *u*_*meas*_ and flow direction *Dir*_*flow*_ are given in Figs. 7 and 8, respectively. In addition, Fig. 7 presents flow velocities calculated by formulas (3) and (4).

The extreme wind parameters recorded in the survey period were as follows: *W*_*sea*_ = 19.93 m/s, *Dir*_*wind*_ = 246.50° (on Feb. 18, 2023). The extreme measured superficial flow velocity amounted to *u*_*meas*_ = 1.09 m/s, with the direction *Dir*_*flow*_ = 310.95° (on Jan. 29, 2024), while the maximum flow velocity calculated by Eqs. (3) and (4) was *u*_*calc*_ = 0.70 m/s (corresponding to the strongest wind observed on Feb. 18, 2023). Several samples in the *u*_*meas*_ series exceed 1 m/s. Figure 7 shows that the calculated extreme velocities are underestimated with respect to the measured ones. Most probably, formulas (3) and (4) reflect long-lasting mild and moderate wind-current conditions better than they do instantaneous stormy wind-current situations, in which the inertia effect does not allow the wind-induced current to develop fully. This underestimation can cause, for example, an inaccuracy in modelling of phenomena driven by the wind-induced currents, e.g. sediment transport. However, the overall statistical analysis shows that the root-mean-square error (*RMSE*) of the modelled flow velocity with respect to the measured signal amounts to 0.11 m/s only.

Figure 6 implies that the predominating wind direction *Dir*_*wind*_ (“from”) ranges from 200° to 250°, while Fig. 8 shows the predominating flow direction *Dir*_*flow*_ (“to”) in the range from 50° to 100°. This suggests that the measured flow can be identified as a wind-driven current with a roughly alongshore direction, i.e. from WSW to ENE (cf. Fig. 1). Again, this finding matches the characteristics of the hydro-meteorological climate identified in the south Baltic Sea.

**Fig. 9:**
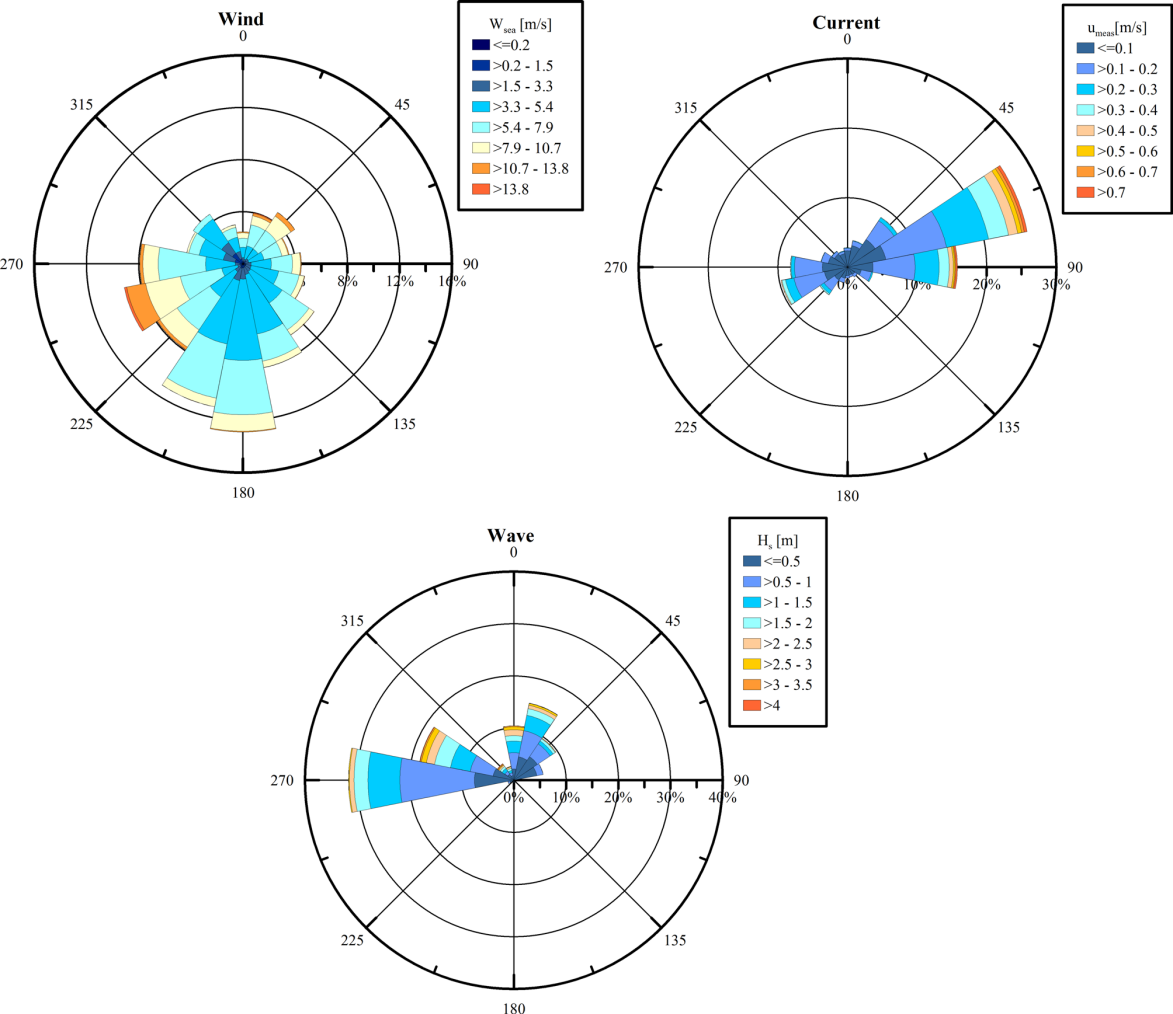
Rose plots of wind (“from”), currents (“to”) and waves (“from”) measured at CRS Lubiatowo in the period from Nov. 26, 2022 to Jan. 30, 2024.

In order to confirm and detail the findings concerning directional features of the investigated phenomena, the rose plots with respect to wind, currents and waves have been produced, see Fig. 9.

The wind rose reveals predominance of wind blowing from S, with the strong and relatively frequent winds from SW-W. The current rose indicates that the most intensive currents are directed towards ENE and E which confirms that the measured flow is mostly a wind-driven current. The wave rose shows the waves coming predominantly from W, with the maximum height *H*_*s*_ of 2–2.5 m. The highest relatively frequent waves, with *H*_*s*_ amounting to 3–3.5 m, are observed from WNW and NNW. Wind waves are a spectral phenomenon so the strongest winds from WSW (see the wind rose) contribute to generation of waves from W. The coastline is located approximately on the direction from WSW to ENE (cf. Fig. 1). Therefore, the winds blowing towards the sea (from directions E-SW) have a small fetch and do not give rise to appearance of any distinct (noticeable) wave motion.

In order to quantify the relations between measured variables (wind, waves and currents) in more detail the Dot Product was used:

$$\text{for wave}-\text{current relations}: Dot Product=\overrightarrow{U}\bullet \overrightarrow{C}=\left|U\right|\bullet \left|C\right|\bullet \text{cos}\left({\theta }_{U}-{\theta }_{C}\right)$$

$$\text{for wind}-\text{current relations}: Dot Product=\overrightarrow{U}\bullet \overrightarrow{W}=\left|U\right|\bullet \left|W\right|\bullet \text{cos}\left({\theta }_{U}-{\theta }_{W}\right)$$

$$\text{for wave}-\text{wind relations}: Dot Product=\overrightarrow{W}\bullet \overrightarrow{C}=\left|W\right|\bullet \left|C\right|\bullet \text{cos}\left({\theta }_{W}-{\theta }_{C}\right)$$

where |*U*| is the current velocity. |*C*| is the wave group velocity (for waves on deep water: $\left|C\right|=\frac{gT}{4\pi }$). |*W*| is the wind velocity. *θ*_*U*_ is the current direction (recalculated to “from” direction). *θ*_*C*_ is the wave direction (from). *θ*_*W*_ is the wind direction (from).

**Fig. 10:**
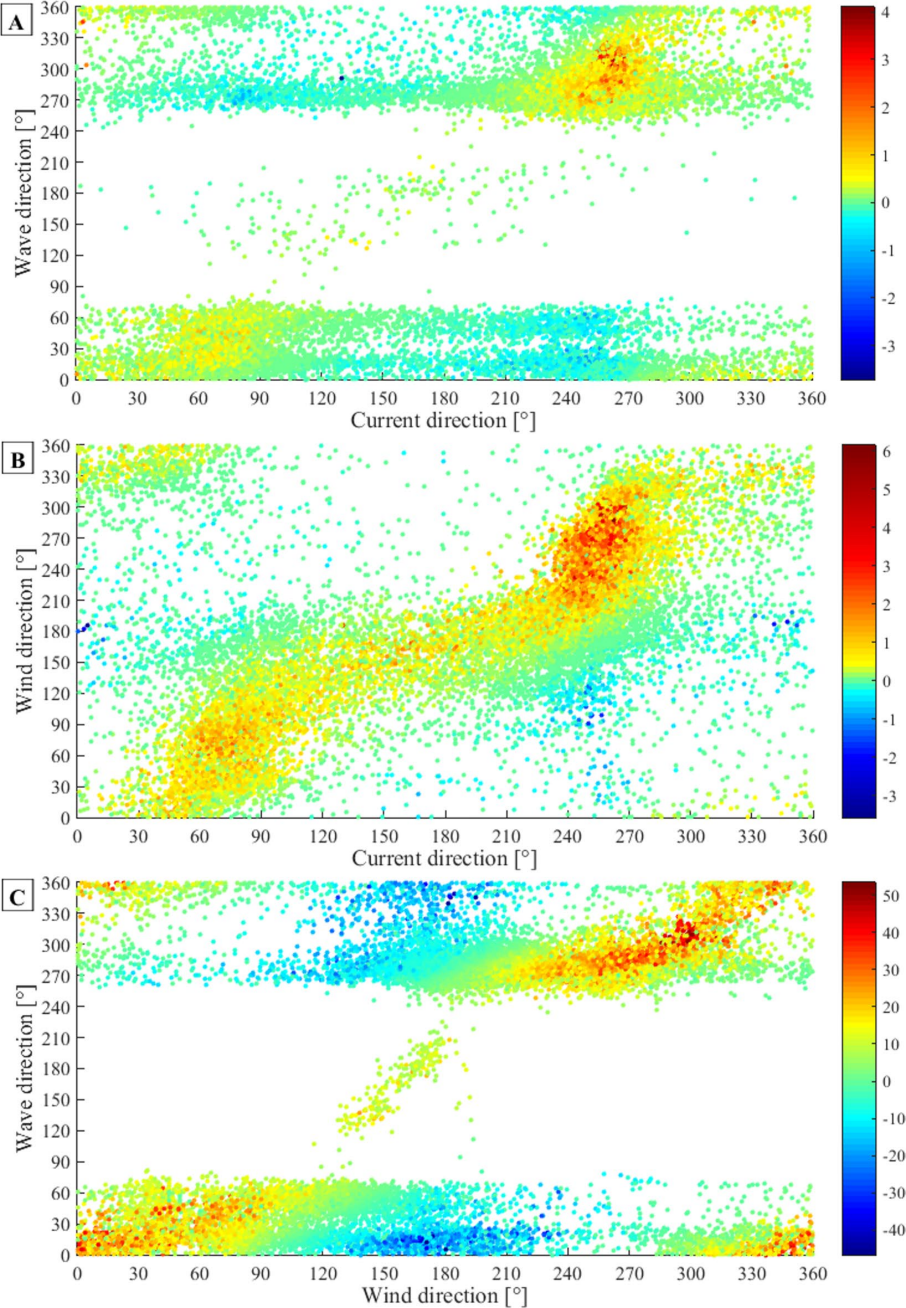
The scatterplots of Dot Products for: (**A**) wave-current relation, (**B**) wind-current relation, (**C**) wave-wind relation.

The calculated Dot Products are presented in Fig. 10. The interpretation is as follows:

1. Positive value: variables in roughly the same direction (the higher value the bigger magnitude of both or one of the variables),
2. value close to zero: variables perpendicular to each other,
3. negative value: variables in opposite directions (the higher value the bigger magnitude of both or one of the variables).

The Dot Product results for wave-current relations (Fig. 10A) reveal the most collinear wave and current directions for waves propagating roughly from W-NW (260°–320°) and water flows from WSW-W (240°–280°) which suggests that both processes are predominantly driven by the same factor (wind). The most collinear winds and currents (Fig. 10B) occur for winds blowing approximately from SSW-NW (200°–315°) and currents from WSW-W (240°–270°). This can support the hypothesis that the wind is the main driving force of the steady water flow in the considered region. Figure 10C shows the most distinct collinearity for waves approaching the site from W-NW (270°–315°) and accompanying (driving) wind from WSW-NW (240°–315°), as well as from N-NW (0°–50°) and NNW-ENE (330°–70°), respectively. The above results are compliant to a large extent with the rose plots shown in Fig. 9.

## Discussion

Situated along Poland’s northern coastline, Lubiatowo occupies a pivotal position in the nation’s evolving energy landscape. Flanked by two transformative energy projects: Poland’s first nuclear power facility^11^ (with a cooling system comprising the intake of cold coastal water and the discharge of warmed water to the sea) to the west and a cluster of offshore wind farms to the northwest, north and northeast (comprising the nearest planned offshore wind farm infrastructures: Baltic Power, C-Wind, Orlen Neptun VIII, Baltica-1, Baltica-2, Baltica-3^18^) Lubiatowo emerges as a focal point where renewable and nuclear energy trajectories converge. This unique geographical juxtaposition not only underscores the region’s strategic importance but also amplifies the necessity for comprehensive environmental assessments, particularly concerning coastal and marine dynamics.

The development and operation of large-scale energy installations in coastal zones demand a nuanced understanding of local hydrodynamic conditions. In Lubiatowo, the interplay of wave activity and surface currents plays a critical role in informing the design, placement, and resilience of energy infrastructure. Accurate measurements of these parameters are essential for mitigating risks associated with coastal erosion, sediment transport, and structural integrity.

Surface current and wave parameter datasets are essential for the safe and efficient operation of offshore wind farms and coastal nuclear power plants. Incorporating detailed hydrodynamic data into the planning and development of energy projects is crucial for ensuring their sustainability and resilience. Notably, high surface current velocities can challenge previous design assumptions, underscoring the importance of these factors in models of sediment transport and dispersion of contingent pollutants. For instance, understanding sediment transport patterns can inform the placement of submarine cables, their burial depth, and the design of protective structures or for nuclear power plants, intake placement of the cooling water system. Moreover, anticipating the effects of extreme wave events and shifting current regimes can guide the implementation of adaptive measures to safeguard infrastructure against climate-induced changes. Understanding local hydrodynamic processes play a critical role in enhancing structural stability, optimizing performance, and informing climate resilience strategies.

In conclusion, Lubiatowo’s strategic location at the intersection of major energy initiatives necessitates a comprehensive approach that integrates hydrodynamic research into energy infrastructure planning. Such integration ensures that developments are not only technologically and economically viable but also environmentally attuned and resilient to the dynamic coastal processes characteristic of the Baltic Sea region.

The presented findings have been achieved using a multi-method approach, comprising viewing of the time series, rose plots and Dot Products. One can deem that this approach can be successfully applied elsewhere, for other coastal regions worldwide.

## Conclusions

The paper presents results of more than a 14-month long field survey monitoring wave-current and wind parameters at a remote foreshore location representative of the south Baltic Sea.

In the survey period (from Nov. 26, 2022 to Jan. 30, 2024), the extreme stormy wave heights recorded on Dec. 23, 2023 amounted to *H*_*s*_ = 4.36 m and *H*_*max*_ = 7.33 m, being smaller by 0.06 m and 0.47 m, respectively, than the maximum values of *H*_*s*_ and *H*_*max*_ ever measured at this location in the past.

The maximum measured superficial flow velocity amounted to *u*_*meas*_ = 1.09 m/s. Several samples in the *u*_*meas*_ series exceed 1 m/s. Such strong superficial currents had never been recorded at this location. The wind-driven superficial flow velocity was modelled by a simple approach yielding a satisfactory agreement with the measured quantities. The calculated extreme velocities are, however, underestimated with respect to the measured ones.

The extreme wind parameters recorded in the survey period were as follows: *W*_*sea*_ = 19.93 m/s, *Dir*_*wind*_ = 246.50°.

The field survey results show that the predominating direction of wave propagation lies on an azimuth of about 275° (approximately from W to E). The highest relatively frequent waves, with *H*_*s*_ amounting to 3–3.5 m, are observed from WNW and NNW. The winds predominantly blow from S but stronger and relatively frequent winds blow from SW-W. The speedy currents are directed mostly towards ENE and E. This implies that the dominant water flow is a wind-driven current with a roughly alongshore direction, i.e. from WSW to ENE. The above results match the characteristics of the hydro-meteorological climate identified in the south Baltic Sea by earlier investigations.

The authors hope that the experimental material presented here advances studies of hydrodynamic processes occurring in the remote foreshore region of non-tidal seas. That coastal area is located beyond the depth of closure—at depths larger than 15 m in the case of the Baltic Sea. The present experimental findings can help determine the forces driving sediment transport and thereby provide a more precise prediction of coastal morphodynamics, including the appearance and behaviour of sandy bed forms. The knowledge of hydro-, litho- and morphodynamic processes in the remote foreshore is useful in planning marine engineering projects, such as wind farms. The presented results, particularly the identification of high surface current velocities, provide new insights that may challenge previous assumptions about the site’s hydrodynamic regime. This finding emphasizes the need to integrate such data into load and scour modeling to enhance the design safety margins of offshore wind mill foundations, cable burial and laying design, as well as nuclear facility cooling systems. Furthermore, understanding the combined effects of waves and currents aids in anticipating climate-driven shifts, supporting adaptive strategies for infrastructure resilience.

## Supplementary Information

Supplementary Information.

## Data Availability

All data generated or analysed during this study are included in this published article and its Supplementary Information files.
